# Shortages and price variability of essential cytotoxic medicines for treating children with cancers

**DOI:** 10.1136/bmjgh-2020-003282

**Published:** 2020-11-10

**Authors:** Yehoda M Martei, Kotoji Iwamoto, Ronald D Barr, John T Wiernkowski, Jane Robertson

**Affiliations:** 1 Hematology – Oncology Division, University of Pennsylvania, Philadelphia, Pennsylvania, USA; 2 Health Technology and Pharmaceuticals Programme, World Health Organization Regional Office for Europe, Copenhagen, Denmark; 3 Pediatric Haematology/Oncology, McMaster University and McMaster Children's Hospital, Hamilton, Ontario, Canada; 4 Clinical Pharmacology, University of Newcastle, Waratah, New South Wales, Australia

**Keywords:** cross-sectional survey, child health, health services research, treatment, cancer

## Abstract

**Introduction:**

Low-income and middle-income countries (LMICs) face the largest burden of mortality from childhood cancers with limited access to curative therapies. Few comparative analyses across all income groups and world regions have examined the availability and acquisition costs of essential medicines for treating cancers in children.

**Methods:**

A cross-sectional survey involved countries in five income groups—low-income (LIC), lower-middle-income (LMC), upper-middle-income (UMC), two high-income country groups (HIC1, HIC2). Physicians and pharmacists reported institutional use, availability, stock outs and prices (brand and generic products) of 34 essential medicines. Price comparisons used US$, applying foreign exchange rates (XR) and purchasing power parity (PPP) adjustments. Medicine costs for treating acute lymphoblastic leukaemia (ALL), Burkitt lymphoma (BL) and Wilms tumour (WT) were calculated (child 29 kg, body surface area 1 m^2^). Comparisons were conducted using non-parametric Kruskal-Wallis tests.

**Results:**

Fifty-eight respondents (50 countries) provided information on medicine use, availability and stock outs, with usable price data from 42 facilities (37 countries). The extent of use of International Society of Paediatric Oncology core and ancillary medicines varied across income groups (p<0.0001 and p=0.0002 respectively). LMC and LIC facilities used fewer medicines than UMC and HIC facilities. UMC and LMC facilities were more likely to report medicines not available or stockouts.

Medicine prices varied widely within and between income bands; generic products were not always cheaper than brand equivalents. PPP adjustment showed relatively higher prices in UMC and LMC facilities for some medicines. Medicine costs were highest in HICs for ALL (p=0.0075 XR; p=0.0178 PPP-adjusted analyses) and WT (p =<0.0001 XR; p=0.0007 PPP-adjusted). Medicine costs for BL were not significantly different.

**Conclusion:**

Problems with the availability of essential medicines, dependable supply chains, confidential medicine prices and wide variability in treatment costs contribute to persistent challenges in the care of children with treatable cancers, especially in LMICs.

**What is already known?**

- The Essential Medicines Working Group of the International Society of Paediatric Oncology (SIOP) proposed a list of essential (core) and ancillary antineoplastic drugs to guide selection and procurement of medicines, particularly in low-income and middle-income countries (LMICs) which have the highest mortality burden from childhood cancers.
- There are limited data on the use, availability, shortages, procurement prices of these essential medicines and treatment costs for common, treatable cancers in children across low-income countries (LICs), lower-middle-income countries (LMCs), upper-middle-income countries (UMCs) and high-income countries (HICs).

**What are the new findings?**

- Use, availability and stockouts of SIOP core and ancillary medicines varied across income groups, with LMC and LIC facilities using fewer medicines, whereas UMC and LMC facilities were more likely to report medicines not available or stockouts.
- Medicine prices varied widely within and between income bands; generic products were not always cheaper than brand equivalents; purchasing power parity adjustment showed relatively higher prices in UMC and LMC facilities for some medicines.
- Medicine costs were highest in HICs but varied widely within and between income groups for acute lymphoblastic leukaemia, Burkitt lymphoma and Wilms tumour.

**What do the new findings imply?**

- Irregular medicine availability, unreliable supply chains, confidential medicine pricing and wide variability in treatment costs contribute to persistent disadvantages for children requiring care for treatable cancers, especially in LMICs.
- Reluctance to provide medicine cost information and/or reliance on list prices limit the usefulness of comparisons of medicine and treatment costs and inhibit progress in ensuring equitable and affordable access to essential cancer medicines and treatments.

## Introduction

Childhood cancers constitute only a small proportion of the global cancer burden, but 84% of them occur in low-income and middle-income countries (LMICs), where nearly 90% of the world’s children live and where access to care and to curative treatments with long-term event-free survival benefit is often limited or non-existent.^1^ There are many challenges to providing access to essential cytotoxic medicines for children with cancer in low-resource settings, including affordability, government underfunding and institutional weaknesses in the pharmaceutical sector for procuring and supplying drugs.^2 3^ The challenges have been compounded in recent years by shortages of key cytotoxic medicines that are the cornerstone of effective treatment of cancers in children.^4^ These medicines are often older, out-of-patent products and, in some cases, there are newer and more expensive alternatives available, although these will be unaffordable in many LMICs.^5^ For other medicines, there are no alternatives, so care is compromised.^6^ Such shortages of cytotoxic medicines are now so commonplace that an ethical framework for dealing with the problem has been proposed.^7^

As defined by WHO, on the supply side: ‘shortage’ occurs when the supply of medicines, health products or vaccines identified as essential by the health system is considered to be insufficient to meet public health and patient needs.^8^ This definition refers only to products that have already been approved and marketed, in order to avoid conflicts with research and development agendas. On the demand side: a ‘shortage’ will occur when demand exceeds supply at any point in the supply chain and may ultimately create a ‘stockout’ at the point of appropriate service delivery to the patient if the cause of the shortage cannot be resolved in a timely manner relative to the clinical needs of the patient. These shortages affect all countries regardless of incomes and healthcare systems.^9^ In high-income countries (HICs) the US Food and Drug Administration and the European Medicines Agency maintain webpages with lists of shortages in current medicines, including cytotoxic agents.^10 11^ A study of such shortages in Belgium and France suggested that the three most prominent determinants were manufacturing difficulties, problems of distribution and supply, and factors related to economic aspects, including the profitability of continued marketing of the product.^12^

To address the burden of disease and access barriers, the Essential Medicines Working Group of the International Society of Paediatric Oncology (SIOP) proposed a list of essential (core) and ancillary antineoplastic drugs which should be available in LMICs.^13^ The WHO Model List of Essential Medicines for Children (EMLc) 2015 also includes several additional supportive care agents for treating children with cancer.^14^ However, few studies have conducted a comparative analysis, across income groups and world regions, on the availability and acquisition costs of these essential medicines for children. Although prior studies have evaluated the availability of these drugs on National Essential Medicines Lists, these lists are often outdated and are not reflective of the actual availability of medicines at the point of care.^15^ Similarly, although the Management Sciences for Health Inc (MSH) provides global data on prices, the current data are outdated, with the most recent information available being 2015.^16^ While there have been a number of studies and reports comparing availability and prices of cancer medicines within and between jurisdictions,^17–23^ these studies have focused on list prices for 31 originator medicines in 18 HICs^17^ and in 10 countries of the South East Asian, Western Pacific and East Mediterranean regions,^18^ drawn on limited data pricing sources within the MSH database,^19^ been restricted to a single country^20^ or focused on patient out-of-pocket costs for cancer medications.^21 22^

There are limited data on price and availability of essential medicines for treating cancers in children, particularly in LMICs^24 25^ which bear a disproportionate burden of mortality from childhood cancers.^26^ Studies have reviewed the costs and cost-effectiveness of treating cancers in children in LMICs,^5^ and assessed the availability and affordability of these medicines in India.^27^ We found no studies assessing availability and comparing prices paid for medicines by institutions providing treatment for childhood cancers across all income groups.

The aim of this study was to assess the use and availability of essential medicines for treating cancers in children, and the nature and extent of shortages, including stockouts, of these antineoplastic medicines in selected LICs, MICs and HICs. In addition, we collected information on the cost of procuring these drugs for the institutions providing cancer care.

## Methods

The study was cross-sectional, involving survey-based data collection from a sample of health professionals at institutions providing cancer care to children across the six WHO geographical regions and five income strata based on the World Bank classification of economies.^28^

The World Bank groups are defined on the basis of gross national income (GNI) per capita calculated using the World Bank Atlas Method.^29^ As of July 2015, low-income economies (LICs) are defined as those with a GNI per capita of US$1045 or less; lower-middle-income economies (LMC) US$1046 but less than US$4125; upper-middle-income economies (UMC) US$4125–US$12,735; and high-income economies (HIC) are those with a GNI per capita of US$12 736 or more. For the purposes of analyses, we used five income groupings, further subdividing high-income economies into those with GNI per capita of US$12 736–US$25 000 (HIC1) and those with GNI per capita greater than $25 000 (HIC2).

There are six WHO regional groupings covering the Americas, Africa, Europe, Eastern Mediterranean region, South East Asian region and the Western Pacific region (http://www.who.int/en/).

We sought to collect data from five institutions in each of the six regions and five income groupings (30 strata), giving a total of 150 institutions. However, in some regions such as Africa, we expected an insufficient number of countries within each income stratum to meet this target. The goal was to collect data from 90 to 100 institutions across the regions and income groups.

### Data collection

The data collection tool was emailed out between May and October 2017. Email reminders were sent at 1 monthly intervals to those confirming participation in the study. Participants were asked to complete a survey (Excel spreadsheet) which had two parts, developed to address the availability and costs of essential cytotoxic medicines for childhood cancer treatment—including 18 medicines (21 products) considered SIOP core medicines,^13^ eight medicines on the SIOP ancillary list and an additional three medicines (five products) from the WHO EMLc 2015.^14^

The first part asked whether the nominated medicine was used in the healthcare facility or institution providing cancer care to children, that is, the medicine is included in one of the treatment protocols used in the facility. We then asked whether the medicine was available today if needed for a patient and, if not, whether it had been subject to stockouts and the duration of any stockouts, categorised as more than 1 month or more than 3 months, within the past 12 months. Medicine supplier referred to the usual source of the medicine such as wholesalers, manufacturers, importers or special government programmes where these exist. Stockout was defined as the complete absence of the medicine at the point of delivery to the patient.^8^ Participants could provide additional comments as needed.

Part 2 of the survey sought information on the acquisition costs of the nominated medicines to the healthcare facility. Respondents were encouraged to answer all questions. However, when there was any information which may be commercial-in-confidence (eg, negotiated prices paid by the institution for the cytotoxic medicines), respondents could leave questions unanswered and relevant cells in the Excel spreadsheet data collection tool empty. For prices, respondents were asked to report the purchase price, paid by the institution providing care, of relevant branded or generic medicines in a specified form and strength, for example, vials of 500 mg cyclophosphamide. In some cases, respondents reported national list prices when it was not possible to obtain institution-specific prices. Prices were reported in local currencies and converted to US$ per unit (vial or tablet) using foreign exchange rates (XR) on 30 June 2017,^30^ and World Bank purchasing power parity (PPP) rates for 2017.^31^ The data collection tool also allowed reporting of an alternative strength of the medicine when the specified strength was not used in that institution. While institutions may benefit from bulk purchases, discounts and bundling of medicines, we wanted to capture information on actual costs to the institution to determine the range of costs of chemotherapeutic agents used in treating cancers in children.

Examples of the data collection tools for shortages of medicines and prices are shown in online supplemental table 1. The initial survey was developed in English and translated into Russian for the former Soviet Union countries and French. Subsequent revisions to simplify the data collection tool were guided by the results of an initial pilot of five respondents in different geographical regions.

10.1136/bmjgh-2020-003282.supp1 Supplementary data

### Participants and recruitment

Participants were paediatric oncologists, haematologists and pharmacists working in cancer care facilities providing treatment services to children. They were identified through their membership of either the SIOP (http://siop-online.org/) or the International Society of Oncology Pharmacy Practitioners (ISOPP http://www.isopp.org/). Both organisations indicated their support in recruiting participants for this study. Potential participants were identified by the six Continental Branch Presidents of SIOP and the Secretary of ISOPP. Participants were selected to ensure adequate representation of the six WHO geographical regions and a spread of World Bank income groups.

Additional participants from under-represented regions were recruited through regional cancer networks such as the African Organisation for Research and Training in Cancer, the Central American Consortium and the Latin American Society of Paediatric Oncology, as well as purposive sampling of providers in regions with low or no responses.

More than one respondent per country was allowed. When there was uncertainty or apparent inconsistency in survey responses, the lead investigators sent follow-up enquiries to the relevant respondents and healthcare facilities to clarify any issues identified. The names of the respondents and healthcare facilities were required to allow follow-up when information provided was incomplete or difficult to interpret. However, data collection forms were deidentified for respondent name and healthcare facility once data cleaning and analysis were completed. Only the country identifier was included and reported in our analysis.

### Statistical analysis

The analyses of the data obtained from this study were carried out using descriptive statistics. Continuous data on availability and price were stratified by World Bank income groupings and statistical significance was analysed using non-parametric rank sum (Kruskal-Wallis) tests. The significance threshold was set at p<0.05.

The proportion of facilities which reported specific individual medicines listed as ‘in use’ was calculated for each medicine. The median proportions of facilities reporting usage of the essential drugs were compared for significant trends across income groups. For medicines which were listed as used in the facility, subsequent analyses of stockouts were carried out using the denominator of only facilities reporting that the medicines were in use.

Price data were obtained from respondents able to provide this information. Prices were compared for generic and originator brands of specific formulations. Kruskal-Wallis tests were used to compare median prices, both XR and PPP-adjusted, for specific drugs and formulations.

An additional disease-based approach was used to estimate the cost of three treatment regimens for which prices were available for all drugs listed within the regimen. The regimens, selected based on recommended disease focus areas in the 2015 revision of the WHO EMLc and including medicines likely to be used in LMICs, were paediatric acute lymphoblastic leukaemia (ALL),^32^ Burkitt lymphoma (BL)^33^ and Wilms tumour (WT).^34^ The regimens, adapted by SIOP for use in LMICs, were not the most intensive treatment protocols but were selected by the WHO Global Initiative for Childhood Cancer for other comparative purposes. Medicines costed for the ALL regimen (high-risk pre-B ALL; regimen 2^32^) were mercaptopurine, cyclophosphamide, cytarabine, doxorubicin, l-asparaginase, methotrexate and vincristine. For BL, we applied the regimen using induction with high dose cyclophosphamide and intrathecal methotrexate and consolidation according to risk group 2.^33^ The protocol for WT was based on a metastatic regimen for preoperative chemotherapy and a three drug regimen for postoperative chemotherapy using doxorubicin, actinomycin D, vincristine.^34^ Consequently, we have a mixture of standard risk and high risk (including metastatic) disease. Costs for prednisolone and dexamethasone were not included as these agents are widely used for other indications and are relatively cheaper medicines that would not substantially affect the price comparisons shown.

We estimated the cost of each complete regimen using prices provided by specific facilities, with the following assumptions of weight 29 kg and body surface area of 1 m^2^. Given the small number of facilities reporting pricing data for all medicines in the regimens, income groups were collapsed into three groups: HIC (encompassing HIC1 and HIC2), UMC and LIC+LMC (encompassing LIC and LMC). Comparisons between the different income groups were reported as median total price per treatment regimen, using XR and PPP-adjusted prices for generic, originator or combinations of these medicines.

Data were analysed using STATA (StataCorp. 2015. Stata Statistical Software: Release 14. StataCorp LP.) and RStudio (V.1.2.5042).

### Patient and public involvement

It was not appropriate to involve patients or the public in the design, or conduct, or reporting, or dissemination plans of our research.

## Results

Data were submitted by 58 respondents from 50 different countries (table 1). Some information on drug availability and stockouts was available from 14 HIC2, five HIC1, 15 UMC, 16 LMC and eight LIC facilities. Some respondents were only able to provide official list prices for medicines or declined to provide pricing information citing commercial-in-confidence arrangements. Usable price data for at least one medicine were available from 42 facilities in 37 countries. Data from 14 HIC (HIC2 and HIC1 combined) 9 UMC and 19 LMC+LIC facilities were included in our price analyses.

**Table 1:** Facilities reporting use of medicines to treat paediatric cancers

Medicine	Income group (no of facilities)	Proportion reporting use Median % (IQR %)
**SIOP core**		
l-asparaginase (i), bleomycin (i), carboplatin (i), cisplatin (i), cyclophosphamide (t), cyclophosphamide (i), cytarabine (i), dacarbazine (i), dactinomycin (i), daunorubicin (i), doxorubicin (i), etoposide (c), etoposide (i), hydroxycarbamide (t/c), ifosfamide (i), mercaptopurine (t), methotrexate (t), methotrexate (i), thioguanine (t), vinblastine (i), vincristine (i)	HIC2 (14)	100 (93–100)
	HIC1 (5)	100 (100–100)
	UMC (15)	100 (87–100)
	LMC (16)	81 (69–81)
	LIC (8)	88 (75–88)
Kruskal-Wallis test	P value <0.0001	
**SIOP Ancillary**		
13-cis retinoic acid (t/c), all-trans retinoic acid ATRA (c), busulphan (t), imatinib (t), irinotecan (i), melphalan (t), topotecan (i), vinorelbine (i)	HIC2 (14)	96 (75–100)
	HIC1 (5)	80 (55–100)
	UMC (15)	83 (70–88)
	LMC (16)	31 (31–41)
	LIC (8)	19 (13–31)
Kruskal-Wallis test	P value=0.0002	
**WHO EMLc**		
Calcium folinate (leucovorin) (i), calcium folinate (leucovorin) (t), filgrastim (i), mesna (t), mesna (i)	HIC2 (14)	93 (86–93)
	HIC1 (5)	100 (80–100)
	UMC (15)	100 (87–100)
	LMC (16)	63 (31–69)
	LIC (8)	50 (50–63)
Kruskal-Wallis test	P value=0.068	

(c) capsule; (i) injection; (t) tablet.

HIC2: Australia, Belgium, Canada, Finland, France, Italy, Japan, New Zealand, Qatar, Spain, UK (two facilities), USA (2).

HIC1: Latvia, Malta, Saudi Arabia, Chile (2).

UMC: Angola, Brazil (2), China, Colombia, Dominican Republic, Georgia, Iran, Iraq, Jordan, Malaysia, Panama, Russian Federation, South Africa, Thailand.

LMC: Armenia, Cameroon (2), Ghana, Guatemala, India, Indonesia, Kenya, Kyrgyzstan, Morocco, Myanmar, Nigeria (2) Sudan, Vietnam (2).

LIC: Ethiopia (2), Haiti, Malawi, Nepal, Rwanda, Senegal, Zimbabwe.

EMLc, Essential Medicines for Children; HIC, high-income country; LIC, low-income country; LMC, lower-middle-income country; SIOP, International Society of Paediatric Oncology; UMC, upper-middle-income country.

### Use of medicines

The proportion of facilities which reported the use of the 34 essential medicines for childhood cancers across the different income group are presented in table 1 and online supplemental table 2. Groups LMC and LIC had no individual medicines which were used across all 24 facilities sampled.

There were statistically significant differences in the reported use of the 21 SIOP core medicines by facilities across the five income groups (p<0.0001) and for the eight SIOP ancillary medicines (p=0.0002). LMCs and LICs were less likely to report use of these medicines in their facilities than HICs and UMCs. While a similar pattern was observed in relation to the five WHO EMLc medicines, the differences reported were not statistically significant (table 1).

The least used SIOP core medicines across all income groups were etoposide tablet/capsule (32/58 facilities, 55%), hydroxycarbamide tablet/capsule (33/58, 57%); and thioguanine tablet (32/58, 55%) (online supplemental table 2).

Among the SIOP ancillary list, the least used medicines were 13-cis retinoic acid tablet/capsule (33/58, 57%), busulphan tablet (19/58, 33%) and melphalan tablet (22/58, 38%). Of the additional WHO EMLc agents, mesna tablet (15/58, 26%) was the least used across all facilities.

### Shortages of medicines

#### Medicines not available today

There were more facilities reporting that medicines were ‘not available today’ in UMC and LMC facilities than HIC1, HIC2 and LIC facilities (table 2). For example, among SIOP core medicines, HIC2 facilities had only one medicine ‘not available today’ in 30% or more facilities—asparaginase injection (31%). In contrast, UMC facilities had three such medicines (cyclophosphamide tablet 33%; etoposide capsule 50%, hydroxycarbamide tablet 30%). LMC facilities had eight such medicines (cyclophosphamide tablet 44%, cytarabine injection 31%, dacarbazine injection 44%, daunorubicin injection 64%, etoposide capsule 80%, methotrexate tablet 31%, thioguanine tablet 80%, vinblastine injection 36%). Etoposide capsule (33%) was the only SIOP core medicine not available in more than 30% of LIC facilities which reported using the medicine.

**Table 2:** Facilities which reported medicine is not available today

	No (%) of facilities reporting medicine ‘not available today’*				
	HIC 2	HIC 1	UMC	LMC	LIC
Total no of facilities	n=14	n=5	n=15	n=16	n=8
**SIOP core**					
Asparaginase injection	4/13 (31)	0/5	4/15 (27)	1/13 (8)	0/7
Bleomycin injection	2/13 (15)	0/5	3/15 (20)	4/14 (29)	0/7
Carboplatin injection	1/14 (7)	0/5	1/15 (7)	2/13 (15)	1/7 (14)
Cisplatin injection	1/14 (7)	0/5	1/15 (7)	3/13 (23)	0/6
Cyclophosphamide tablet	1/12 (8)	0/3	4/12 (33)	4/9 (44)	1/6 (17)
Cyclophosphamide injection	1/14 (7)	0/5	2/15 (13)	1/13 (8)	0/7
Cytarabine injection	1/14 (7)	0/5	0/15	4/13 (31)	1/7 (14)
Dacarbazine injection	1/12 (8)	0/5	3/15 (20)	4/9 (44)	0/6
Dactinomycin injection	1/14 (7)	0/5	2/15 (13)	2/12 (17)	1/7 (14)
Daunorubicin injection	1/14 (7)	0/5	2/13 (15)	7/11 (64)	1/5 (20)
Doxorubicin injection	1/14 (7)	0/5	1/15 (7)	3/13 (23)	0/7
Etoposide capsule	2/12 (17)	0/2	5/10 (50)	4/5 (80)	1/3 (33)
Etoposide injection	1/14 (7)	0/5	1/15 (7)	2/14 (14)	0/7
Hydroxycarbamide tablet/capsule	0/10	0/3	3/10 (30)	0/7	0/3
Ifosfamide injection	1/14 (7)	0/5	1/15 (7)	3/12 (25)	1/4 (25)
Mercaptopurine tablet	0/14	0/5	2/14 (14)	3/14 (21)	1/7 (14)
Methotrexate tablet	0/14	0/5	3/13 (23)	4/13 (31)	0/7
Methotrexate injection	0/14	0/5	1/15 (7)	2/14 (14)	0/7
Thioguanine tablet	1/11 (9)	0/4	3/11 (27)	4/5 (80)	0/1
Vinblastine injection	1/14 (7)	0/5	3/15 (20)	4/11 (36)	1/6 (17)
Vincristine injection	1/14 (7)	0/5	2/15 (13)	3/14 (21)	1/7 (14)
**SIOP ancillary**					
13-cis retinoic acid tablet/capsule	2/12 (17)	0/4	4/11 (36)	3/5 (60)	0/1
All-trans retinoic acid capsule	2/13 (15)	0/4	4/13 (31)	3/8 (38)	2/4 (50)
Busulphan tablet	2/5 (40)	0/2	5/9 (56)	1/3 (33)	
Imatinib tablet	1/14 (7)	0/5	4/15 (27)	4/10 (40)	1/6 (17)
Irinotecan injection	1/14 (7)	1/5 (20)	3/14 (21)	2/5 (40)	0/2
Melphalan tablet	1/6 (17)	0/2	2/7 (29)	2/5 (40)	1/2 (50)
Topotecan injection	1/14 (7)	0/5	3/12 (25)	3/5 (60)	0/1
Vinorelbine injection	2/14 (14)	0/3	2/13 (15)	3/6 (50)	0/1
**Other WHO EMLc**					
Calcium folinate (leucovorin) inject	1/13 (8)	0/5	2/15 (13)	1/11 (9)	0/7
Calcium folinate (leucovorin) tablet	1/12 (8)	0/4	4/13 (31)	2/5 (40)	1/5 (20)
Filgrastim injection	1/13 (8)	0/5	1/15 (7)	1/10 (10)	0/4
Mesna tablet	1/6 (17)	0/3	3/6 (50)		
Mesna injection	1/14 (7)	0/5	1/15 (7)	4/13 (31)	0/4

*Only facilities reporting that the medicine is used are included in the denominator for calculation of %.

EMLc, Essential Medicines for Children; HIC, high-income country; LIC, low-income country; LMC, lower-middle-income country; SIOP, International Society of Paediatric Oncology; UMC, upper-middle-income country.

These patterns of more reports of medicines ‘not available today’ in UMC and LMC facilities also applied to SIOP ancillary medicines, with three of eight medicines in UMC facilities and all eight medicines in LMC facilities reported ‘not available today’ in 30% or more facilities which used the medicine (table 2).

#### Medicines not available from suppliers

More facilities in UMCs and LMCs reported that medicines were not available from suppliers, and that suppliers were unable to provide products for more than 1 month and more than 3 months than in HIC or LIC facilities (online supplemental tables 3, 4 and 5).

Asparaginase was the only drug reported as out of stock for more than 1 month in HICs (online supplemental table 4). In contrast, 19 of the 21 SIOP core medicines and all eight SIOP ancillary medicines were reported out of stock for more than 1 month and often more than 3 months in at least one of the UMC facilities that used the medicine. (online supplemental tables 4 and 5).

### Prices of medicines

Given the small number of facilities reporting usable pricing data, analyses were conducted using three groups (HIC combining HIC1 and HIC2, UMC, LMC+LIC). The median prices, in USD XR and PPP-adjusted, are listed for specific generic and originator medicines in online supplemental table 6.

In the XR analysis, there were statistically significant differences in prices across the income groups for eight medicines—asparaginase (originator brand), dactinomycin 500 µg (generic), daunorubicin 20 mg (generic), ifosfamide 1 g (generic), mercaptopurine 50 mg (originator), methotrexate 500 mg (generic), vincristine 1 mg (originator) and vincristine 2 mg (generic) (table 3). However, small numbers in some income strata limits the power of these analyses.

**Table 3:** Median and range of prices for selected SIOP core medicines (foreign exchange (XR) and purchasing price parity (PPP) adjusted)

Medicine	Income group	Originator price			Generic price		
		No prices	Median XR (range)	Median PPP-adjusted (range)	No prices	Median XR (range)	Median PPP-adjusted (range)
Asparaginase injection 10 000 units	HIC	8	133.32 (41.14–1037.76)	143.65 (45.10–1082.58)	5	83.17 (69.20–1113.09)	113.02 (69.57–1676.81)
	UMC	3	33.00 (24.42–109.19)	118.50 (39.92–176.19)	4	73.26 (40.59–100.73)	163.67 (110.50–298.52)
	LMC+LIC	11	35.00 (4.28–83.43)	131.41 (11.64–264.08)	8	33.02 (10.35–156.14)	98.08 (26.79–253.72)
	P value*		0.0102	0.5101		0.0792	0.2691
Dactinomycin injection 500 μg	HIC	7	80.08 (4.52–1351.57)	111.92 (5.07–1351.57)	5	142.49 (62.00–230.33)	142.39 (98.59–239.62)
	UMC	3	103.00 (5.91–140.40)	312.84 (9.67–369.85)	4	37.34 (19.39–48.50)	75.46 (57.48–107.52)
	LMC+LIC	6	13.92 (2.30–24.20)	41.09 (29.97–68.71)	9	12.30 (6.69–46.63)	30.34 (20.98–87.01)
	P value		0.0996	0.4127		0.0020	0.0029
Daunorubicin injection 20 mg	HIC	8	20.57 (4.24–144.51)	31.62 (5.79–144.41)	5	69.10 (21.21–88.12)	71.88 (32.60–88.12)
	UMC	3	17.01 (12.80–20.36)	45.36 (27.81–45.96)	4	24.77 (12.80–35.65)	47.73 (36.04–61.60)
	LMC+LIC	4	8.08 (5.73–34.80)	35.74 (17.98–88.09)	6	4.42 (0.49–8.12)	12.75 (0.80–22.34)
	P value		0.5002	0.9191		0.0052	0.0060
Doxorubicin injection 50 mg	HIC	4	28.59 (7.77–75.65)	37.49 (10.62–82.94)	10	9.20 (6.16–45.16)	14.15 (6.16–49.51)
	UMC	2	74.19 (15.69–132.70)	193.46 (25.65–361.27)	6	16.87 (5.80–338.46)	40.24 (13.44–553.39)
	LMC+LIC	8	13.51 (6.00–26.10)	40.05 (16.23–98.15)	9	12.42 (3.46–31.30)	30.34 (8.96–76.64)
	P value		0.1945	0.8305		0.8159	0.0209
Ifosfamide injection 1 g	HIC	8	33.23 (18.18–81.46)	39.97 (29.70–92.06)	6	65.70 (12.62–102.00)	64.52 (20.61–166.60)
	UMC	4	28.68 (11.49–36.38)	74.84 (18.79–99.06)	4	12.10 (10.02–22.00)	26.51 (16.39–49.28)
	LMC+LIC	7	16.00 (5.73–95.70)	76.36 (17.98–242.25)	5	7.73 (4.78–47.98)	28.07 (12.79–77.96)
	P value		0.335	0.7577		0.0318	0.3091
Mercaptopurine tablet 50 mg	HIC	7	3.15 (0.63–3.70)	3.53 (0.63–7.62)	7	1.45 (0.05–3.14)	1.41 (0.06–4.74)
	UMC	6	0.92 (0.05–3.28)	1.50 (0.14–7.32)	3	0.30 (0.03–0.50)	0.67 (0.08–1.49)
	LMC+LIC	9	0.21 (0.01–4.66)	0.68 (0.02–11.80)	9	0.12 (0.03–1.54)	0.42 (0.04–3.62)
	P value		0.0342	0.4268		0.0821	0.398
Vincristine injection 1 mg	HIC	3	15.49 (6.67–25.69)	15.48 (10.89–28.17)	7	5.06 (3.37–45.00)	6.70 (4.60–73.50)
	UMC	3	5.80 (2.94–23.87)	20.83 (4.81–38.52)	5	5.77 (4.49–8.00)	13.31 (9.44–17.92)
	LMC+LIC	8	1.86 (0.25–10.44)	8.80 (2.70–26.43)	10	2.21 (0.70–74.52)	5.73 (1.76–121.09)
	P value		0.0337	0.2998		0.2178	0.2069

*Kruskal-Wallis non-parametric test.

HIC, high-income country; LIC, low-income country; LMC, lower-middle-income country; SIOP, International Society of Paediatric Oncology; UMC, upper-middle-income country.

Only three medicines showed statistically significant differences in the PPP-adjusted analyses—two in common with the XR analyses, dactinomycin 500 µg (generic) and daunorubicin 20 mg (generic)—and doxorubicin 50 mg (generic).

#### Range of prices

There was often a wide range of prices within an income band. For example, prices for asparaginase 10 000 units injection in HIC ranged from US$41.14–US$1037.76 for originator brand (prices from eight facilities) and US$69.20–US$1113.09 for the generic product (five facilities) (table 3). This also likely reflects use of the different asparaginase products in different facilities—native asparaginase derived from *Escherichia coli* (asparaginase), a pegylated form of this enzyme (PEG-asparaginase) and a product isolated from Erwinia chrysanthemi, Erwinia asparaginase, the most expensive formulation for which six doses are required to replace one dose of PEG-asparaginase.^35^ The much narrower range of prices in UMC and LMC+LIC countries may reflect use of the less expensive asparaginase product(s) in these facilities.

Generic products were not consistently cheaper than their originator brand equivalents. For example, in HIC, the median prices (XR) for generic dactinomycin, daunorubicin and ifosfamide were higher than for the originator products, although the range of prices was generally wider for the originator product.

PPP adjustment illustrates the relatively higher prices paid by UMC and LMC+LIC for some medicines. The median PPP-adjusted price for originator asparaginase was higher in LMC+LIC (US$131.41) than in UMC (US$118.50) and neither was substantially lower than the median price paid in HIC (US$143.65). Median PPP-adjusted prices for daunorubicin, doxorubicin and ifosfamide originator products were lowest in HIC (table 3).

### Costs of treatment regimens

**Figure 1:**
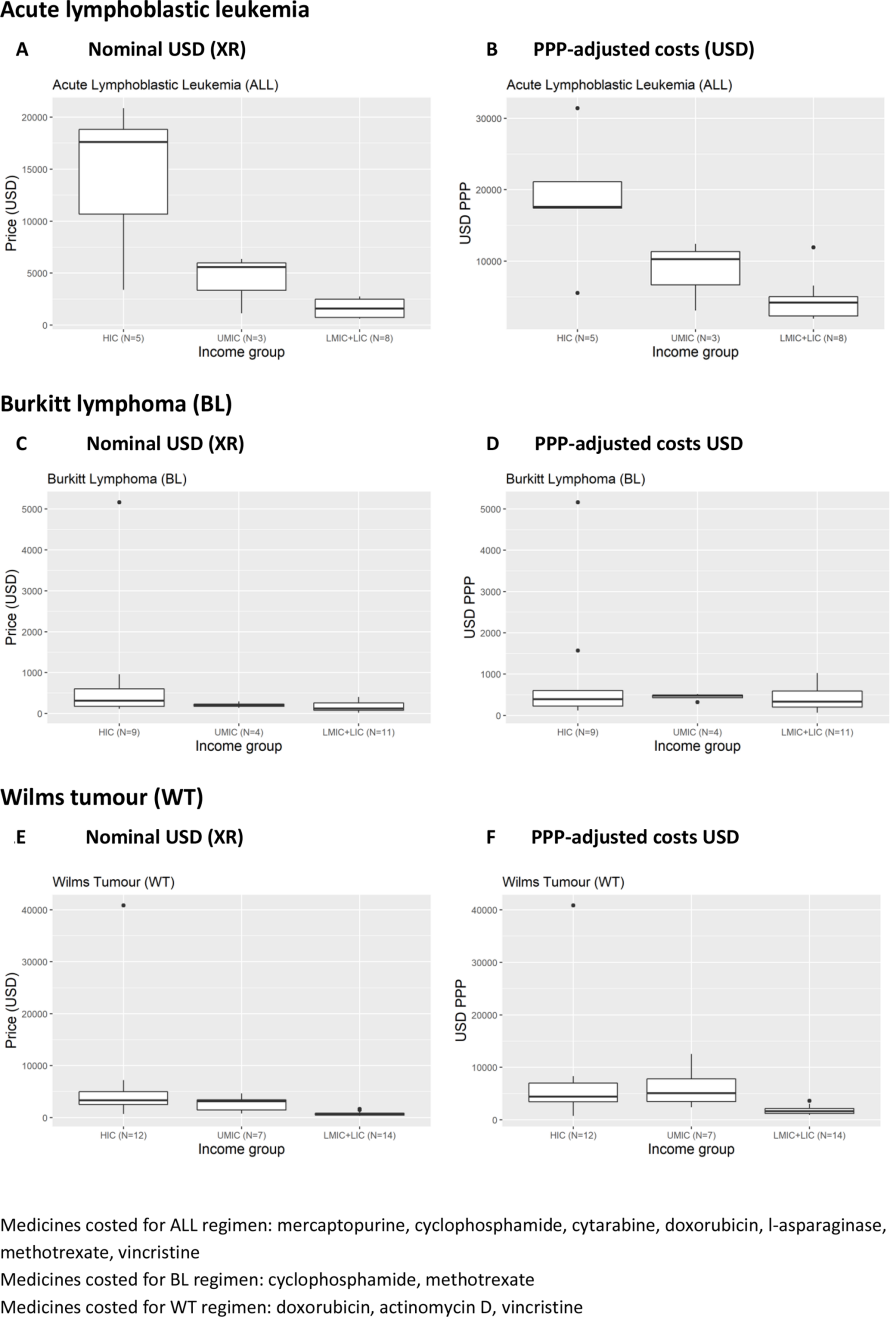
Median, IQR and range of prices for the treatment of acute lymphoblastic leukaemia (ALL), BL and WT. HIC, high-income country; LIC, low-income country; LMIC, low-middle-income country; PPP, purchasing power parity; UMIC, upper-middle-income country.

The costs of treatment for paediatric ALL, BL and WT are shown in online supplemental table 7 and figure 1A–F.

For ALL, the cost of treatment was significantly higher in HIC compared with UMC and LMC+LIC facilities for both nominal USD (XR) and PPP-adjusted analyses (p=0.0075 and p=0.0178, respectively). There was a wide range of treatment costs within each income band. For example, costs ranged from US$3391.46–US$20 859.91 (XR analysis) and US$5539.44–US$31 424.26 (PPP-adjusted analysis) across five HIC facilities and US$618.85–US$2754.65 (XR analysis) and US$1942.00–US$11 943.86 (PPP-adjusted analysis) across eight LMC and LIC facilities (online supplemental table 7).

There were no statistically significant differences across income groups of BL treatment costs with XR and PPP-adjusted analyses. However, as with ALL, there were wide variations in treatment costs within income groups. Across 11 LMC+LIC facilities treatment costs ranged from US$20.44–US$405.80 (XR analysis) and US$64.13–US$1027.24 (PPP-adjusted analysis)

Treatment costs for WT could be derived for 33 facilities (12 HIC, 7 UMC, 14 LMC+LIC), with costs significantly higher in HIC compared with UMC and LMC+LIC facilities for both nominal USD (XR) and PPP-adjusted analyses (p ≤0.0001 and p=0.0007, respectively). As with ALL and BL, there was a wide range of treatment costs within each income band. For example, costs ranged from US$673.10–US$40 859.26 (XR analysis) and US$755.05–US$40 859.26 (PPP-adjusted analysis) across 12 HIC facilities and US$307.51–US$1631.40 (XR analysis) and US$940.77–US$3643.18 (PPP-adjusted analysis) across 14 LMC+LIC facilities.

## Discussion

In this cross-sectional study, we evaluated the scope of essential medicines for childhood cancers listed in use in facilities within 50 countries encompassing different WHO world regions and income groups, as well as the availability of these medicines for clinical use at the point of care. Consistent with prior studies, our analyses showed significant variability in medicine availability and higher rates of suboptimal access in LMICs compared with HICs^22 24^ as well as variability in prices within countries and between countries of similar economic status.^17 36–38^

Furthermore, in a detailed assessment of medicine prices and corresponding prices of treatment regimens for common paediatric cancers, our comparative price analysis highlights the significant variability in the cost of treatment across and within different income groups, even for generic formulations. Generic medicines were not always cheaper than the innovator product, perhaps reflecting manufacturer discounts and rebates for brand-name products, loss-leader pricing and bundled purchasing of some products, or small numbers of generic manufacturers and little competition.^39 40^ Official list prices do not take account of confidential discounts and rebates.^23^ Difficulties with acquisition of data on prices also highlight challenges with addressing problems with prices if there is lack of transparency and no reliable data to describe the extent of the problem.^36 41^ At the World Health Assembly in May 2019, Member States adopted a resolution (WHA72.8) to improve the transparency of markets for medicines, vaccines, and other health products.^42^

In our initial assessment of drugs listed in use, SIOP core medicines were the most likely group to be listed in use in facilities across all income groups. However, the proportion of facilities listing these medicines in use was significantly higher in high-income and middle-income settings. Although these medicines were listed in use, UMCs were more likely, along with LMCs and LICs to list these medicines as being unavailable and out of stock with suppliers. This may reflect more efficient supply lines and larger buffer stocks held in HIC institutions. By performing these two analyses we capture not only the listing of a drug in use but provide data on the ability of a patient at the point of care to receive these drugs from the specific facilities.

There are several possible reasons for lower levels of reported availability of key medicines in LMCs than LICs. LICs may use a narrower range of clinical protocols or manage lower stages of disease and therefore rely on a smaller number of agents. This is consistent with the observations that LICs use fewer SIOP ancillary and WHO EMLc medicines than LMCs. More LICs may also be part of twinning arrangements with cancer care centres in HICs that facilitate access to medicines. Some LICs reported donor programmes supplying medicines at reduced cost to LIC facilities. These external supply mechanisms may overcome some of the challenges of national centralised procurement processes and limited government budgets for purchase of cytotoxic medicines.

Stockout was most prevalent in UMCs, LMCs and LICs, especially for SIOP ancillary medicines for which a median of 40% of facilities reported stockout in LMC facilities. Of note, however, 31% of HIC2 facilities reported stockout of asparaginase, one of the key medicines in treating paediatric ALL, which is the most common childhood cancer with a survival rate of 90% for patients who complete specified treatment regimens in HICs.^43^ These analyses highlight global issues with paediatric cancer drug stockout most prevalent in UMCs, LMCs and LICs, but specific key drug shortages in HICs could pose significant challenges and result in inferior survival outcomes for childhood cancer patients.^4^

The mechanisms for drug shortages and stockouts may be different across income groups and it is likely that both international and local disruptions contribute to vulnerabilities in the supply chain.^15 25^ Our results show that, for drugs which are out of stock at the time of the survey in UMCs, LMCs and LICs, suppliers being out of stock, sometimes for more than 3 months, contribute to extended stock out periods of specific drugs. HICs are also affected, with shortages of vincristine, methotrexate and Erwinia asparaginase threatening delivery of care for children with ALL.^44–46^ In some cases these shortages are attributed to decrease in production because of low profits associated with the manufacturing of generic formulations.^46–49^

Few studies have examined the prices of essential medicines for specific diseases within individual countries.^27 50 51^ This study adds to the current literature by performing a detailed examination of the price of essential medicines for childhood cancers across different income groups and world regions. Similar to prior data on medicine prices for adult cancers^41^ and MSH prices,^16^ this study showed significant heterogeneity in the costs for treating two of the three most common cancers. Differences in median treatment costs between HIC, UMC and LMC+LIC facilities were much smaller when expressed in PPP-adjusted costs, with median costs being higher in UMCs than HICs for BL and WT. However, costs for treating BL in HIC and UMC will be underestimated as we applied costs to a treatment regimen that would mostly be used in LICs. It is difficult to directly compare treatment costs with other studies that have included medical and non-medical costs in addition to pharmaceutical costs.^5 52^ However, we are reassured that our country estimate of ALL treatment costs was broadly similar to that calculated by Faruqui *et al* 27 using a similar but not identical treatment regimen and recognising wide variations in prices of different brands of the same anticancer drug in the same dose and dosage form manufactured in India.^37^

This study has several limitations. It is based on the accuracy of self-reports provided by health professionals in different facilities and could be subject to inaccuracies. We believe that the expertise of the participants and their interest in the problem being investigated ensures the collection of valid and reliable data. Furthermore, the lead study investigators had interim meetings to review data quality and discrepancies. When the data were discrepant, follow-up enquiries were made to reach the relevant respondent and healthcare facilities to clarify any issues identified. Another limitation of this study is the challenge of comparing different formulations of the same drug across income groups and countries. For instance, asparaginase in HICs is unlikely to be the same native product used in LMCs and LICs, with differences in products noted even within Europe.^36 53^ The special issue with asparaginase is compounded by the marketing and distribution in LMICs of substandard products^54^ which have deleterious effects on children with ALL.^55^ Significant additional challenges were apparent in obtaining price data. The response rates were much lower on price data, with some facilities unable to provide data due to commercial-in-confidence arrangements and agreements with group purchasing organisations.^56^

The limited sample size may misrepresent the true access to chemotherapy in individual countries but may be accurate for LMICs where there are few paediatric cancer centres. However, it may be inaccurate for HICs where substantial regional heterogeneity exists.^24^ Therefore, we are cautious about generalising price data for a single institution as representative of the whole country. Finally, these prices do not take into consideration public/private insurance coverage and out-of-pocket expenses or some drug access programmes available to patients. Moreover, lower prices in certain LMICs may not reflect affordability for patients if 100% of drugs are covered out-of-pocket by families.^21 22 41 57^ The data are still important for highlighting variability in price regulatory structures and costs for treatment in different countries, even for generic formulations.

Despite these limitations, the study has several strengths. We are unaware of any other studies which have measured the comparative scope of the prices and costs in different regions of the world and in different economic settings. By bringing together information on the extent of the problems and potential cost implications of shortages of drugs for the care of children with cancers, we aim to heighten awareness and promote discussions on practical solutions to ensure that essential, effective and affordable cytotoxic medicines continue to be marketed and available. Traditional donor funding is unlikely to be a useful mechanism for providing access to cytotoxic medicines in LMICs.^58 59^ However, mechanisms such as those used by GAVI (The Vaccine Alliance), with Advance Market Commitments which commit to purchase quality-assured products at negotiated prices, may encourage manufacturers to plan for longer periods knowing that demand exists and there is a sustainable business case with a guaranteed affordable long-term price.^60^ WHO will have an important role to play in advancing these discussions along with professional societies such as SIOP and ISOPP.

Many of the issues we have identified here are not new and confirm the persistent disadvantage for children requiring care for treatable cancers, especially in LMICs. Medicine costs are only one aspect of cancer care provision. However, irregular medicine availability, unreliable supply chains, high prices relative to GNI per capita, confidential medicine pricing that conceals benefits to governments able to negotiate effectively with industry, out-of-pocket costs to patients and their families, and limited government budgets to purchase cytotoxic medicines all contribute to problems in providing care. Documenting relative disadvantage is a starting point, but reluctance to provide medicine cost information and/or reliance on list prices severely limit the usefulness of comparisons of medicine and treatment costs and inhibit progress in ensuring equitable and affordable access to essential cancer medicines and treatments.

## Data Availability

No additional data are available.
