# Supplementary material for: Shortages and price variability of essential cytotoxic medicines for treating children with cancers
Source: BMJ Glob Health. 2020 Nov 10;5(11):e003282. doi: 10.1136/bmjgh-2020-003282 (PMC7656942; doi:10.1136/bmjgh-2020-003282)
Supplement: Supplementary data [file bmjgh-2020-003282supp001.pdf]

SUPPLEMENTARY TABLES

Supplementary Table 1: Examples of data collection for medicines shortages (1a) and prices (1b)

1a. Medicines shortages

	Medicine of interest	Used in this facility?[1] SEE FOOTNOTE	In stock and available today	Could order today and stock available from Supplier[2] SEE FOOTNOTE	Supplier[2] currently out of stock	Supplier[2] out of stock for more than one month during past 12 months	Supplier[2] out of stock for more than three months during past 12 months	Any additional comments on issues of shortage of this medicine
	Response options	Yes / No	Yes / No	Yes / No	Yes / No	Yes / No	Yes / No	Free text responses
SIOP Core list of essential medicines	Asparaginase injection							
	Bleomycin injection							
	Carboplatin injection							
	Cisplatin injection							
	Cyclophosphamide tablet							
	Cyclophosphamide injection							
	Cytarabine injection							
	Dacarbazine injection							
	Dactinomycin injection							
	Daunorubicin injection							
	Doxorubicin injection							
	Etoposide capsule							
	Etoposide injection							

1b. Medicines acquisition prices

		Originator				Generic					
	Medicine	Number of units per pack		Acquisition price to hospital or clinic in local currency		Number of units per pack		Acquisition price to hospital or clinic in local currency		If the product with this strength is not used, is there alternative strength used in this facility?	Any additional comments on issues of shortage of this medicine
	Response options	Number	unit	NUMBERS ONLY	Currency	Number	unit	NUMBERS ONLY	Currency	Strength, number of units per pack, price per pack, originator or generic	Free text responses
	Example	5	vial/amp	88.5	USD	10	tab	40	USD	50mg, 1 vial/pack, 50 USD/pack, originator	
SIOP Core list of essential medicines	Asparaginase injection 10 000 Units		vial/amp				vial/amp				
	Bleomycin injection 15 mg		vial/amp				vial/amp				
	Carboplatin injection 150 mg		vial/amp				vial/amp				
	Cisplatin injection 100 mg		vial/amp				vial/amp				
	Cyclophosphamide tablet 25 mg		tab				tab				
	Cyclophosphamide injection 500 mg		vial/amp				vial/amp				
	Cytarabine injection 100 mg		vial/amp				vial/amp				
	Cytarabine injection 1 gram		vial/amp				vial/amp				
	Dacarbazine injection 100 mg		vial/amp				vial/amp				
	Dactinomycin injection 500 micrograms		vial/amp				vial/amp				

Supplementary Table 2: Facilities which reported medicine is used in the facility

	Number (%) of facilities reporting using the medicine				
	**HIC 2**	**HIC 1**	**UMC**	**LMC**	**LIC**
**Total number of facilities**	**n=14**	**n=5**	**n=15**	**n=16**	**n=8**
**SIOP CORE**					
Asparaginase injection	13 (93)	5 (100)	15 (100)	13 (81)	7 (88)
Bleomycin injection	13 (93)	5 (100)	15 (100)	14 (88)	7 (88)
Carboplatin injection	14 (100)	5 (100)	15 (100)	13 (81)	7 (88)
Cisplatin injection	14 (100)	5 (100)	15 (100)	13 (81)	6 (75)
Cyclophosphamide tablet	12 (86)	3 (60)	12 (80)	9 (56)	6(75)
Cyclophosphamide injection	14 (100)	5 (100)	15 (100)	13 (81)	7 (88)
Cytarabine injection	14 (100)	5 (100)	15 (100)	13 (81)	7 (88)
Dacarbazine injection	12 (86)	5 (100)	15 (100)	9 (56)	6 (75)
Dactinomycin injection	14 (100)	5 (100)	15 (100)	12 (75)	7 (88)
Daunorubicin injection	14 (100)	5 (100)	13 (87)	11 (69)	5 (63)
Doxorubicin injection	14 (100)	5 (100)	15 (100)	13 (81)	7 (88)
Etoposide capsule	12 (86)	2 (40)	10 (67)	5 (31)	3 (38)
Etoposide injection	14 (100)	5 (100)	15 (100)	14 (88)	7 (88)
Hydroxycarbamide tablet/capsule	10 (71)	3 (60)	10 (67)	7 (44)	3 (38)
Ifosfamide injection	14 (100)	5 (100)	15 (100)	12 (75)	4 (50)
Mercaptopurine tablet	14 (100)	5 (100)	14 (93)	14 (88)	7 (88)
Methotrexate tablet	14 (100)	5 (100)	13 (87)	13 (81)	7 (88)
Methotrexate injection	14 (100)	5 (100)	15 (100)	14 (88)	7 (88)
Thioguanine tablet	11 (79)	4 (80)	11 (73)	5 (31)	1 (13)
Vinblastine injection	14 (100)	5 (100)	15 (100)	11 (69)	6 (75)
Vincristine injection	14 (100)	5 (100)	15 (100)	14 (88)	7 (88)
**SIOP ANCILLARY**					
13-cis retinoic acid tablet/capsule	12 (86)	4 (80)	11 (73)	5 (31)	1 (13)
All-trans retinoic acid ATRA capsule	13 (93)	4 (80)	13 (87)	8 (50)	4 (50)
Busulphan tablet	5 (36)	2 (40)	9 (60)	3 (19)	0
Imatinib tablet	14 (100)	5 (100)	15 (100)	10 (63)	6 (75)
Irinotecan injection	14 (100)	5 (100)	14 (93)	5 (31)	2 (25)
Melphalan tablet	6 (43)	2 (40)	7 (47)	5 (31)	2(25)
Topotecan injection	14 (100)	5 (100)	12 (80)	5 (31)	1 (13)
Vinorelbine injection	14 (100)	3 (60)	13 (87)	6 (38)	1(13)
**OTHER WHO EMLc**					
Calcium folinate (leucovorin) injection	13 (93)	5 (100)	15 (100)	11 (69)	7 (88)
Calcium folinate (leucovorin) tablet	12 (86)	4 (80)	13 (87)	5 (31)	5 (63)
Filgrastim injection	13 (93)	5 (100)	15 (100)	10 (63)	4 (50)
Mesna tablet	6 (43)	3 (60)	6 (40)	0	0
Mesna injection	14 (100)	5 (100)	15 (100)	13 (81)	4 (50)

HIC high-income country, UMC upper-middle-income country, LMC lower-middle-income country, LIC low-income country

Supplementary Table 3: Facilities where medicine unavailable from supplier

	Number (%) of facilities where medicine unavailable from supplier				
	**HIC 2**	**HIC 1**	**UMC**	**LMC**	**LIC**
**Total number of facilities**	**n=14**	**n=5**	**n=15**	**n=16**	**n=8**
**SIOP CORE**					
Asparaginase injection	1 / 4 (25)		2 / 4 (50)	0 / 1	
Bleomycin injection	0 / 2		2 / 3 (67)	1 / 4 (25)	
Carboplatin injection	0 / 1		1 / 1 (100)	0 / 2	1 / 1 (100)
Cisplatin injection	0 / 1		1 / 1 (100)	0 / 3	
Cyclophosphamide tablet	0 / 1		2 / 4 (50)	0 / 4	1 / 1 (100)
Cyclophosphamide injection	0 / 1		1 / 2 (50)	0 / 1	
Cytarabine injection	0 / 1			0 / 4	1 / 1 (100)
Dacarbazine injection	0 / 1		2 / 3 (67)	2 / 4 (50)	
Dactinomycin injection	0 / 1		1 / 2 (50)	1 / 2 (50)	1 / 1 (100)
Daunorubicin injection	0 / 1		2 / 2 (100)	4 / 7 (57)	1 / 1 (100)
Doxorubicin injection	0 / 1		0 / 1	0 / 3	
Etoposide capsule	0 / 2		3 / 5 (60)	2 / 4 (50)	1 / 1 (100)
Etoposide injection	0 / 1		1 / 1 (100)	0 / 2	
Hydroxycarbamide tablet/capsule			2 / 3 (67)		
Ifosfamide injection	0 / 1		1 / 1 (100)	0 / 3	1 / 1 (100)
Mercaptopurine tablet			1 / 2 (50)	2 / 3 (67)	1 / 1 (100)
Methotrexate tablet			1 / 3 (33)	0 / 4	
Methotrexate injection			1 / 1 (100)	1 / 2 (50)	
Thioguanine tablet	0 / 1		1 / 3 (33)	4 / 4 (100)	
Vinblastine injection	0 / 1		3 / 3 (100)	2 / 4 (50)	1 / 1 (100)
Vincristine injection	0 / 1		2 / 2 (100)	1 / 3 (33)	1 / 1 (100)
**SIOP ANCILLARY**					
13-cis retinoic acid tablet/capsule	0 / 2		2 / 4 (50)	1 / 3 (33)	
All-trans retinoic acid ATRA capsule	0 / 2		2 / 4 (50)	1 / 3 (33)	2 / 2 (100)
Busulphan tablet	0 / 2		2 / 5 (40)	1 / 1 (100)	
Imatinib tablet	0 / 1		1 / 4 (25)	2 / 4 (50)	1 / 1 (100)
Irinotecan injection	0 / 1	0 / 1	3 / 3 (100)	2 / 2 (100)	
Melphalan tablet	0 / 1		1 / 2 (50)	2 / 2 (100)	1 / 1 (100)
Topotecan injection	0 / 1		2 / 3 (67)	2 / 3 (67)	
Vinorelbine injection	0 / 2		1 / 2 (50)	3 / 3 (100)	
**OTHER WHO EMLc**					
Calcium folinate (leucovorin) inject	0 / 1		0 / 2	0 / 1	
Calcium folinate (leucovorin) tablet	0 / 1		1 / 4 (25)	0 / 2	1 / 1 (100)
Filgrastim injection	0 / 1		0 / 1	0 / 1	
Mesna tablet	0 / 1		1 / 3 (33)		
Mesna injection	0 / 1		1 / 1 (100)	0 / 4	

HIC high-income country, UMC upper-middle-income country, LMC lower-middle-income country, LIC low-income country

Supplementary Table 4: Facilities reporting that supplier is out of stock for more than one month in past 12 months

	Number (%) of facilities where medicine out of stock > one month				
	**HIC 2**	**HIC 1**	**UMC**	**LMC**	**LIC**
**Total number of facilities**	**n=14**	**n=5**	**n=15**	**n=16**	**n=8**
**SIOP CORE**					
Asparaginase injection	1 / 1 (100)		2 / 2 (100)		
Bleomycin injection			2 / 2 (100)	1 / 1 (100)	
Carboplatin injection			1 / 1 (100)		0 / 1
Cisplatin injection			1 / 1 (100)		
Cyclophosphamide tablet			2 / 2 (100)		1 / 1 (100)
Cyclophosphamide injection			1 / 1 (100)		
Cytarabine injection					1 / 1 (100)
Dacarbazine injection			2 / 2 (100)	1 / 2 (50)	
Dactinomycin injection			1 / 1 (100)	0 / 1	1 / 1 (100)
Daunorubicin injection			2 / 2 (100)	3 / 4 (75)	0 / 1
Doxorubicin injection					
Etoposide capsule			3 / 3 (100)	2 / 2 (100)	1 / 1 (100)
Etoposide injection			1 / 1 (100)		
Hydroxycarbamide tablet/capsule			1 / 2 (50)		
Ifosfamide injection			1 / 1 (100)		1 / 1 (100)
Mercaptopurine tablet			1 / 1 (100)	1 / 2 (50)	1 / 1 (100)
Methotrexate tablet			1 / 1 (100)		
Methotrexate injection			1 / 1 (100)	1 / 1 (100)	
Thioguanine tablet			1 / 1 (100)	2 / 4 (50)	
Vinblastine injection			3 / 3 (100)	2 / 2 (100)	1 / 1 (100)
Vincristine injection			2 / 2 (100)	1 / 1 (100)	1 / 1 (100)
**SIOP ANCILLARY**					
13-cis retinoic acid tablet/capsule			2 / 2 (100)	1 / 1 (100)	
All-trans retinoic acid ATRA capsule			2 / 2 (100)	1 / 1 (100)	1 / 2 (50)
Busulphan tablet			1 / 2 (50)	0 / 1	
Imatinib tablet			1 / 1 (100)	1 / 2 (50)	1 / 1 (100)
Irinotecan injection			2 / 3 (67)	0 / 2	
Melphalan tablet			1 / 1 (100)	2 / 2 (100)	0 / 1
Topotecan injection			2 / 2 (100)	1 / 2 (50)	
Vinorelbine injection			1 / 1 (100)	2 / 3 (67)	
**OTHER WHO EMLc**					
Calcium folinate (leucovorin) inject					
Calcium folinate (leucovorin) tablet			1 / 1 (100)		0 / 1
Filgrastim injection					
Mesna tablet			0 / 1		
Mesna injection			1 / 1 (100)		

HIC high-income country, UMC upper-middle-income country, LMC lower-middle-income country, LIC low-income country

Supplementary Table 5: Facilities reporting that supplier is out of stock for more than three months in past 12 months

	Number (%) of facilities where medicine out of stock > three months				
	**HIC 2**	**HIC 1**	**UMC**	**LMC**	**LIC**
**Total number of facilities**	**n=14**	**n=5**	**n=15**	**n=16**	**n=8**
**SIOP CORE**					
Asparaginase injection	0 / 1		1 / 2 (50)		
Bleomycin injection			1 / 2 (50)	1 / 1 (100)	
Carboplatin injection			1 / 1 (100)		0 / 1
Cisplatin injection			1 / 1 (100)		
Cyclophosphamide tablet			2 / 2 (100)		1 / 1 (100)
Cyclophosphamide injection			1 / 1 (100)		
Cytarabine injection					1 / 1 (100)
Dacarbazine injection			2 / 2 (100)	1 / 2 (50)	
Dactinomycin injection			1 / 1 (100)	0 / 1	1 / 1 (100)
Daunorubicin injection			2 / 2 (100)	2 / 4 (50)	0 / 1
Doxorubicin injection					
Etoposide capsule			3 / 3 (100)	2 / 2 (100)	1 / 1 (100)
Etoposide injection			1 / 1 (100)		
Hydroxycarbamide tablet/capsule			1 / 2 (50)		
Ifosfamide injection			1 / 1 (100)		1 / 1 (100)
Mercaptopurine tablet			1 / 1 (100)	1 / 2 (50)	1 / 1 (100)
Methotrexate tablet			1 / 1 (100)		
Methotrexate injection			1 / 1 (100)	1 / 1 (100)	
Thioguanine tablet			1 / 1 (100)	2 / 4 (50)	
Vinblastine injection			2 / 3 (67)	2 / 2 (100)	1 / 1 (100)
Vincristine injection			2 / 2 (100)	1 / 1 (100)	0 / 1
**SIOP ANCILLARY**					
13-cis retinoic acid tablet/capsule			2 / 2 (100)	1 / 1 (100)	0 / 0
All-trans retinoic acid ATRA capsule			2 / 2 (100)	1 / 1 (100)	1 / 2 (50)
Busulphan tablet			0 / 2	0 / 1	0 / 0
Imatinib tablet			1 / 1 (100)	1 / 2 (50)	1 / 1 (100)
Irinotecan injection			2 / 3 (67)	0 / 2	
Melphalan tablet			1 / 1 (100)	2 / 2 (100)	0 / 1
Topotecan injection			2 / 2 (100)	1 / 2 (50)	
Vinorelbine injection			1 / 1 (100)	2 / 3 (67)	
**OTHER WHO EMLc**					
Calcium folinate (leucovorin) inject					
Calcium folinate (leucovorin) tablet			1 / 1 (100)		0 / 1
Filgrastim injection					
Mesna tablet			0 / 1		
Mesna injection			1 / 1 (100)		

HIC high-income country, UMC upper-middle-income country, LMC lower-middle-income country, LIC low-income country

Supplementary Table 6: Median prices for originator brand and generic product using foreign exchange (XR) and purchasing price parity (PPP) adjustment

Medicine	Income group	Originator price			Generic price		
		No. of prices	Median XR	Median PPP	No. of prices	Median XR	Median PPP
**Asparaginase injection 10 000 Units**	HIC	8	133.32	143.65	5	83.17	113.02
	UMIC	3	33.00	118.50	4	73.26	163.67
	LMIC+LIC	11	35.00	131.41	8	33.02	98.08
	*p-value**		**0.0102**	0.5101		0.0792	0.2691
**Bleomycin injection 15 mg**	HIC	5	18.15	24.81	9	31.32	44.64
	UMIC	3	25.61	69.71	4	38.48	66.87
	LMIC+LIC	7	19.90	43.77	10	12.82	39.05
	p-value		0.2706	0.129		0.0815	0.3286
**Carboplatin injection 150 mg**	HIC	2	89.80	117.98	10	11.11	14.66
	UMIC	3	26.73	61.76	4	17.53	46.28
	LMIC+LIC	8	40.00	120.34	8	10.72	28.47
	p-value		0.1541	0.9729		0.5743	0.639
**Cisplatin injection 100 mg**	HIC	1	27.27	44.55	10	15.42	15.90
	UMIC	2	7.28	11.81	1	3.54	10.49
	LMIC+LIC	6	10.00	32.60	5	7.48	19.35
	p-value		0.1992	0.1133		0.2098	0.8626
**Cyclophosphamide injection 500 mg**	HIC	6	9.93	13.00	4	27.02	44.13
	UMIC	2	9.68	19.83	2	6.77	15.12
	LMIC+LIC	9	4.20	13.53	8	7.22	13.00
	p-value		0.1476	0.7411		0.1408	0.298
**Cyclophosphamide tablet 25 mg**	HIC	1	0.32	0.52	4	0.74	0.80
	UMIC	0	NA	NA	0	NA	NA
	LMIC+LIC	3	1.00	2.71	1	0.52	1.26
	p-value		0.6547	0.1797		> 0.9999	0.4795
**Cytarabine injection 1 gram**	HIC	4	20.44	34.76	9	19.58	22.93
	UMIC	5	26.07	58.09	2	13.57	31.90
	LMIC+LIC	6	14.55	54.51	3	14.33	44.95
	p-value		0.2281	0.7018		0.4479	0.86
**Cytarabine injection 100 mg**	HIC	5	7.58	12.37	8	2.61	3.29
	UMIC	6	3.95	10.61	1	3.97	10.80
	LMIC+LIC	7	4.33	15.10	6	2.65	9.05
	p-value		0.7315	0.2389		0.8688	0.2395
**Dacarbazine injection 100 mg**	HIC	3	22.74	25.51	6	12.26	16.50
	UMIC	1	4.75	17.07	5	13.75	22.98
	LMIC+LIC	5	13.44	37.30	2	14.61	39.01
	p-value		0.1894	0.278		0.9772	0.6666
**Dactinomycin injection 500 micrograms**	HIC	7	80.08	111.92	5	142.49	142.39
	UMIC	3	103.00	312.84	4	37.34	75.46
	LMIC+LIC	6	13.92	41.09	9	12.30	30.34
	p-value		0.0996	0.4127		**0.0020**	**0.0029**

Medicine	Income group	Originator price			Generic price		
		No. of prices	Median XR	Median PPP	No. of prices	Median XR	Median PPP
**Daunorubicin injection 20 mg**	HIC	8	20.57	31.62	5	69.10	71.88
	UMIC	3	17.01	45.36	4	24.77	47.73
	LMIC+LIC	4	8.08	35.74	6	4.42	12.75
	p-value		0.5002	0.9191		**0.0052**	**0.0060**
**Daunorubicin injection 50 mg**	HIC	2	513.42	737.46	0	NA	NA
	UMIC	0	NA	NA	0	NA	NA
	LMIC+LIC	3	20.00	60.93	1	9.55	29.97
	p-value		0.0833	0.0833		NA	NA
**Doxorubicin injection 10 mg**	HIC	2	12.48	13.78	8	3.89	4.91
	UMIC	3	19.81	35.71	5	3.34	7.01
	LMIC+LIC	4	3.26	10.91	7	2.87	8.99
	p-value		0.0863	0.3012		0.58	0.4179
**Doxorubicin injection 50 mg**	HIC	4	28.59	37.49	10	9.20	14.15
	UMIC	2	74.19	193.46	6	16.87	40.24
	LMIC+LIC	8	13.51	40.05	9	12.42	30.34
	p-value		0.1945	0.8305		0.8159	**0.0209**
**Etoposide capsule 100 mg**	HIC	3	6.68	7.55	0	NA	NA
	UMIC	0	NA	NA	1	7.56	22.41
	LMIC+LIC	1	4.00	19.63	1	1.65	4.26
	p-value		0.1797	0.1797		0.3173	0.3173
**Etoposide injection 100 mg**	HIC	3	30.89	30.87	13	5.77	6.43
	UMIC	2	5.15	14.09	7	6.00	13.44
	LMIC+LIC	6	7.00	19.59	10	5.26	12.50
	p-value		0.0661	0.3909		0.8693	0.4774
**Hydroxycarbamide tablet/capsule 250 mg**	HIC	2	0.75	1.15	2	0.51	0.76
	UMIC	1	0.01	0.02	1	0.50	0.81
	LMIC+LIC	1	0.22	0.68	1	0.41	1.01
	p-value		0.2592	0.4066		0.8607	0.8607
**Ifosfamide injection 1gram**	HIC	8	33.23	39.97	6	65.70	64.52
	UMIC	4	28.68	74.84	4	12.10	26.51
	LMIC+LIC	7	16.00	76.36	5	7.73	28.07
	p-value		0.335	0.7577		**0.0318**	0.3091
**Mercaptopurine tablet 50 mg**	HIC	7	3.15	3.53	7	1.45	1.41
	UMIC	6	0.92	1.50	3	0.30	0.67
	LMIC+LIC	9	0.21	0.68	9	0.12	0.42
	p-value		**0.0342**	0.4268		0.0821	0.398
**Methotrexate injection 50 mg**	HIC	3	7.58	12.37	10	3.37	5.70
	UMIC	4	5.62	10.31	4	3.48	9.80
	LMIC+LIC	8	1.63	7.90	8	3.18	6.07
	p-value		0.1775	0.9404		0.9133	0.2674
**Methotrexate Injection 500 mg**	HIC	1	10.61	17.32	6	15.23	17.78
	UMIC	2	42.64	68.88	3	16.71	27.32

Medicine	Income group	Originator price			Generic price		
		No. of prices	Median XR	Median PPP	No. of prices	Median XR	Median PPP
	LMIC+LIC	6	5.75	15.55	7	2.87	8.99
	p-value		0.1171	0.6703		**0.0451**	0.1262
**Methotrexate tablet 2.5 mg**	HIC	6	0.16	0.21	8	0.09	0.14
	UMIC	3	0.16	0.30	5	0.13	0.25
	LMIC+LIC	5	0.50	1.54	8	0.08	0.26
	p-value		0.5334	0.37		0.5236	0.4939
**Thioguanine tablet 40 mg**	HIC	8	6.65	7.90	5	3.69	3.43
	UMIC	5	1.88	3.07	2	1.68	4.95
	LMIC+LIC	1	2.18	5.51	2	0.24	0.85
	p-value		0.3593	0.6858		0.1979	0.3114
**Vinblastine injection 10 mg**	HIC	4	22.31	23.59	9	13.01	20.11
	UMIC	2	12.07	31.09	6	17.44	41.87
	LMIC+LIC	6	10.95	28.61	4	6.08	18.43
	p-value		0.1678	0.9025		0.1708	0.0895
**Vincristine injection 1 mg**	HIC	3	15.49	15.48	7	5.06	6.70
	UMIC	3	5.80	20.83	5	5.77	13.31
	LMIC+LIC	8	1.86	8.80	10	2.21	5.73
	p-value		**0.0337**	0.2998		0.2178	0.2069
**Vincristine injection 2 mg**	HIC	0	NA	NA	6	9.15	10.55
	UMIC	0	NA	NA	1	11.99	26.71
	LMIC+LIC	3	0.78	2.00	4	2.04	5.17
	p-value		NA	NA		**0.0286**	0.0618

*Kruskal-Wallis non-parametric test

Supplementary Table 7 Range of medicine prices for treatment of selected paediatric cancers in children in nominal USD (XR) and purchasing price parity (PPP) adjusted

Income group*	Number of facilities	Cost of treatment regimen			
		Nominal USD (XR)		PPP-adjusted USD	
		Median	Range	Median	Range
**Acute lymphoblastic leukemia (ALL)**					
HIC	5	$7,599.14	$3391.46 – $20,859.91	$17,587.09	$5,539.44 – $31,424.26
UMC	3	$5,572.44	$1,133.05 – $6,367.06	$10,273.87	$3,084 - $12,416,17
LIC+LMC	8	$1,597.79	$618.85 – $2,754.65	$4,180.90	$1,942.00 – $11,943.86
Kruskal Wallis		p = 0.0075		p = 0.0178	
**Burkitt Lymphoma (BL)**					
HIC	9	$314.85	$115.51 – $5,161.87	$395.25	$120.17 – $5,161.87
UMC	4	$199.66	$143.52 – $299.58	$474.66	$319.78 – $520.90
LIC+LMC	11	$121.00	$20.44 – $405.80	$334.50	$64.13 – $1027.24
Kruskal Wallis		p = 0.0905		p = 0.8678	
**Wilms Tumour (WT)**					
HIC	12	$3,296.01	$673.10 – $40,859.26	$4,423.43	$755.05 – $40,859.26
UMC	7	$3,115.87	$807.87 - $4,635.83	$5,094.58	$2,394.16 - $12,560.49
LIC+LMC	14	$636.78	$307.51 - $1,631.40	$1,670.83	$940.77 - $3,643.18
Kruskal Wallis		p < 0.0001		p = 0.0007	

HIC high-income country, UMC upper-middle-income country, LMC lower-middle-income country, LIC low-income country

Medicines costed for ALL regimen: mercaptopurine, cyclophosphamide, cytarabine, doxorubicin, l-asparaginase, methotrexate, vincristine

Medicines costed for BL regimen: cyclophosphamide, methotrexate

Medicines costed for WT regimen: doxorubicin, actinomycin D, vincristine
